# Retracted: Bacterial Biodegradation of Crude Oil Using Local Isolates

**DOI:** 10.1155/2016/6013871

**Published:** 2016-03-03

**Authors:** International Journal of Bacteriology

The article titled “Bacterial Biodegradation of Crude Oil Using Local Isolates” [1] has been retracted as it was found to be substantially similar to the following previously published article: “Biodegradation Of Crude Oil Using Local Isolates,” by Aboelwafa, A. M., and Alwasify, R. S., published in Australian Journal of Basic and Applied Sciences.
